# Molecular Subtyping Based on EGFR Mutation‐Associated Genes and the Prognostic Role of TRAF2 in Lung Adenocarcinoma

**DOI:** 10.1155/humu/1664289

**Published:** 2026-06-10

**Authors:** Bo Gao, Zihan Dong, Taotao Yan, Zhiwang Liu, Jianle Chen

**Affiliations:** ^1^ Department of Thoracic Surgery, Affiliated Hospital of Nantong University, Medical School of Nantong University, Nantong, China, ahnmc.com; ^2^ Suzhou Medical College, Soochow University, Suzhou, China, scu.edu.tw; ^3^ Department of Pediatric Intensive Care Unit, Children Hospital of Soochow University, Suzhou, China

**Keywords:** bioinformatics, EGFR, germline mutation, lung adenocarcinoma, somatic mutation, TRAF2

## Abstract

This study is aimed at systematically identifying key genes associated with EGFR mutations and developing a molecular classification model in lung adenocarcinoma (LUAD) using integrative bioinformatics approaches. Multi‐omics datasets derived from cBioPortal and The Cancer Genome Atlas (TCGA) LUAD cohort were interrogated to identify genes correlated with EGFR mutational status. A core set of 18 genes exhibiting significant associations with both EGFR mutation frequency and patient prognosis was identified. Based on this gene signature, a two‐cluster molecular subtype stratification was established. These subtypes demonstrated statistically significant divergence in overall survival, immune cell infiltration profiles, and predicted responsiveness to immunotherapeutic intervention. Further analyses, including machine learning algorithms, multivariate Cox regression, and molecular docking, identified TRAF2 as a key prognostic gene closely associated with EGFR. In vitro experiments demonstrated that TRAF2 promotes proliferation, migration, and invasion of LUAD cells. Additional analyses suggested that TRAF2 may contribute to tumor progression through epigenetic regulation and associated signaling pathways. Collectively, these findings provide novel insights into the molecular heterogeneity of EGFR‐mutant LUAD and offer potential targets for precision prognostic assessment and combination therapeutic strategies.

## 1. Introduction

Lung cancer remains the leading cause of cancer incidence and cancer‐related mortality worldwide [1]. Among its subtypes, lung adenocarcinoma (LUAD) represents the predominant histological subtype of non‐small cell lung cancer (NSCLC) [2]. In recent years, comprehensive treatment strategies—including surgical resection, conventional chemotherapy and radiotherapy, molecular targeted therapy, and immunotherapy—have made substantial clinical progress and significantly improved overall survival in a subset of patients with LUAD [3]. However, LUAD is characterized by pronounced molecular heterogeneity and a highly complex tumor microenvironment (TME). Patients frequently develop tumor invasion, distant metastasis, and secondary drug resistance during the mid‐to‐late stages of treatment. Notably, with the widespread application of targeted therapies and immune checkpoint inhibitors, acquired resistance driven by mechanisms such as metabolic reprogramming and immune evasion has emerged as a critical bottleneck limiting long‐term patient survival [4–7]. In view of these persisting clinical challenges, there exists a compelling imperative to elucidate the fundamental molecular mechanisms orchestrating LUAD initiation and progression, to refine the identification of precise prognostic biomarkers, and to investigate innovative multitarget therapeutic paradigms.

Epidermal growth factor receptor (EGFR) is frequently mutated in LUAD, including both somatic and germline alterations, which play critical roles in lung cancer development. EGFR mutations exhibit strong clinical relevance in LUAD, particularly among East Asian populations, where their prevalence is markedly higher [8]. Somatic mutations, such as Exon 19 deletions and the L858R point mutation in Exon 21, represent the most common variants and are closely associated with sensitivity to EGFR tyrosine kinase inhibitors (TKIs) [9, 10]. In addition, although relatively rare, germline EGFR mutations may also contribute to lung cancer susceptibility. For instance, the germline T790M mutation has been implicated in hereditary predisposition to LUAD, with a notable prevalence among never‐smokers [11, 12]. Mechanistically, germline EGFR mutations may promote tumorigenesis by enhancing autophosphorylation of the EGFR protein [12]. Beyond their role in tumor initiation, EGFR mutational status is tightly correlated with clinical outcome. Multiple lines of evidence attest to a significant association between EGFR mutation carriage and both disease‐free survival and overall survival in LUAD cohorts. Notably, within the context of early‐stage disease, patients harboring EGFR mutations generally exhibit a more favorable prognosis relative to those bearing wild‐type EGFR [13, 14]. However, EGFR mutations are often accompanied by co‐occurring genetic alterations, such as TP53 and RB1 mutations, which may confer resistance to EGFR‐TKI therapy and are associated with poorer clinical outcomes [15, 16]. The heterogeneity and complexity of EGFR mutations further complicate therapeutic management. Compound EGFR mutations, defined as the presence of multiple concurrent alterations within the EGFR gene, are generally associated with inferior clinical outcomes [17, 18]. Moreover, intratumoral somatic heterogeneity in *EGFR* mutational status may underpin variable therapeutic responses and the eventual emergence of acquired drug resistance [19, 20]. Therefore, comprehensive molecular profiling is essential for identifying patients who are most likely to benefit from precision medicine approaches [21]. In summary, EGFR mutations in LUAD—including both somatic and germline alterations—play crucial roles in tumor initiation and progression. A deeper understanding of their molecular mechanisms and clinical implications is essential for optimizing therapeutic strategies and improving patient outcomes [22, 23].

Bioinformatics plays a pivotal role in the development of diagnostic and prognostic biomarkers in oncology [24–27]. In light of the aforementioned background and clinical challenges, this study is aimed at systematically identifying key genes highly associated with EGFR mutations through genome‐wide bioinformatics analyses and evaluating their roles in the prognosis and immune microenvironment of LUAD. To achieve this, we integrated multidimensional cohort data from large‐scale public databases, including cBioPortal and TCGA‐LUAD. Through comprehensive analysis, we identified 18 core genes significantly associated with both EGFR mutation frequency and patient prognosis. Based on these genes, we further established a novel molecular clustering classification. This classification effectively revealed significant differences among patient subtypes in terms of clinical outcomes, immune cell infiltration patterns, and responsiveness to immunotherapy. Moreover, through multivariate regression analysis, molecular docking, and functional enrichment analysis, TRAF2 was identified as a key prognostic gene closely interacting with EGFR. Importantly, in vitro cellular experiments further demonstrated, for the first time, the direct role of TRAF2 in promoting the proliferation, migration, and invasion of LUAD cells. Collectively, this study provides new insights into the molecular network dynamics underlying EGFR‐mutant LUAD and highlights promising molecular targets for the development of combination therapeutic strategies aimed at overcoming drug resistance, as well as for the construction of precise prognostic models in clinical practice.

## 2. Materials and Methods

### 2.1. Datasets and Patient Samples

We used samples from the “Lung Adenocarcinoma Met Organotropism (MSK, Cancer Cell 2023)” dataset in the cBioPortal database to analyze the mutation frequency of EGFR and its association with the prognosis of patients with LUAD. Additionally, high‐frequency mutated genes in both the EGFR‐mutant and EGFR‐non‐mutant groups were derived from the same dataset. We also included 539 LUAD samples and 59 normal samples from the TCGA‐LUAD dataset. The methylation level of TRAF2 was analyzed using the Shiny Methylation Analysis Resource Tool (SMART) database [28].

### 2.2. Non‐Negative Matrix Factorization (NMF) Cluster Analysis

We applied NMF to identify meaningful biological coefficients from the evaluated gene expression matrix. Utilizing this technique allowed us to effectively arrange both samples and genes, thereby highlighting the underlying structural characteristics of our dataset. Focusing on these intrinsic properties facilitated sample categorization, ultimately yielding deeper insights into the underlying biological interactions and distinct patterns among the studied cohorts [29]. Subsequently, we utilized the “Limma” software package in R to perform differential expression analysis, aiming to investigate transcriptional variations among distinct groups, with a particular focus on comparing cluster A against cluster B. To guarantee the selection of highly significant transcriptional alterations, we set strict filtering thresholds: an adjusted *p* value of less than 0.05 alongside a log‐fold change (|log*F* *C*|) magnitude greater than 0.5. Following this preliminary assessment, we re‐applied the “NMF” tool in R to categorize the entire cohort anew, relying exclusively upon those differentially expressed genes (DEGs) discovered during the subcluster comparison. The objective behind this subsequent classification was to uncover latent molecular subtypes, providing clearer perspectives regarding the active biological mechanisms driving the data. During this computational phase, we implemented the “brunet” method. We conducted 100 iterations per defined group size, testing possible cluster counts spanning from *k* = 2 up to 10. To ascertain the optimal quantity of subgroups, we carefully assessed multiple validation parameters, specifically dispersion, silhouette width, and cophenetic correlation.

### 2.3. Immune Infiltration and Molecular Docking

To achieve a high‐fidelity quantification of immune scores, our study leveraged the R‐based “immunedeconv” toolkit [30]. The various computational frameworks embedded in this package have been subjected to extensive validation, with each providing specific analytical strengths. Furthermore, we executed molecular docking simulations via the CB‐Dock2 platform, having retrieved the three‐dimensional configuration of gemcitabine and osimertinib from the PubChem repository [31].

### 2.4. Cell Culture

The LUAD lines A549 (Cat# CL‐0016) and NCI‐H1299 (Cat# CL‐0165) were sourced from Procell Life Science & Technology. A549 cells were cultivated in F‐12K medium (iCell, Cat# iCell‐0007), whereas the NCI‐H1299 line was maintained in RPMI‐1640 (MilliporeSigma, Cat# R8758). Both culture media were supplemented with 10% fetal bovine serum (FBS) and 1% penicillin–streptomycin (P/S). All cultures were kept in a high‐humidity incubator at 37°C with a 5% CO_2_ atmosphere.

### 2.5. Quantitative Real‐Time PCR (qRT‐PCR)

Total cellular RNA was isolated using Trizol reagent, followed by cDNA synthesis using the RevertAid FirstStrand kit. We executed the qRT‐PCR protocol on an Applied Biosystems 7900HT Fast Real‐Time PCR platform, adhering to previously described reaction settings. The specific primer pairs utilized for target genes were as follows: TRAF2 (Forward: 5 ^′^‐ TGGCTGGCCGCATACC‐3 ^′^, Reverse: 5 ^′^‐ TGTAGCCGTACCTGCTGGTGTA‐3 ^′^) and *β*‐actin (Forward: 5 ^′^‐ AGGATTCCTATGTGGGCGAC ‐3 ^′^, Reverse: 5 ^′^‐ ATAGCACAGCCTGGATAGCAA ‐3 ^′^).

### 2.6. Colony Formation Assay

Following seeding into six‐well plates, cells were maintained in culture for a duration of 14 days. Upon completion of the growth period, colonies were fixed with methanol and subsequently stained using a 0.1% crystal violet solution. Visualization and enumeration of stained colonies were performed utilizing an Olympus microscope.

### 2.7. Transwell Assay

To evaluate cellular invasion and motility, we employed Transwell dishes (Corning Inc.) both with and without Matrigel coating. In brief, transfected colon cancer cells (2 × 10^4^) were resuspended in 100 *μ*L of serum‐deprived medium (Gibco) and loaded into the upper chamber. Meanwhile, the lower compartment was filled with 500 *μ*L of DMEM supplemented with 10% serum (Shanghai ExCell Biology Inc.). After a 24‐h incubation at 37°C in 5% CO_2_, the cells were fixed using 4% paraformaldehyde (Beyotime) for 10 min at ambient temperature. Subsequently, cells were stained for 10 min with 0.2%–0.5% crystal violet (Sigma‐Aldrich) and examined for statistical analysis via an inverted optical microscope (Shanghai Optical Instrument Factory). The migration assay procedure was identical to the invasion assay, excluding the use of Matrigel.

### 2.8. Statistical Analysis

The Wilcoxon rank‐sum test was performed to compare TRAF2 expression between LUAD tissues and normal lung samples. For the assessment of patient prognosis, the log‐rank test was utilized. Results were considered statistically significant if the *p* value was under 0.05.

## 3. Result

### 3.1. Screening of EGFR Mutation‐Related Genes

**Figure 1:**
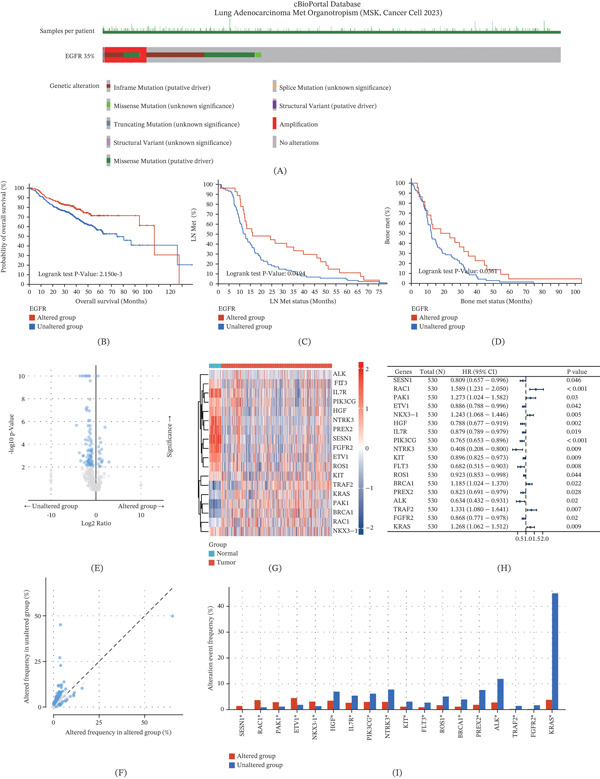
Identification of 18 key differentially expressed and prognostic genes associated with EGFR mutations. (A) Mutation frequency of EGFR in LUAD. (B–D) Prognostic differences between EGFR‐mutant and non‐mutant groups. (E–F) Differential analysis between EGFR‐mutant and non‐mutant groups. (G) Heatmap of the differential expression of key mutant genes. (H) Prognostic significance of the key mutant genes. (I) Mutation frequencies of the key genes between EGFR‐mutant and non‐mutant groups.

In the evolutionary landscape of LUAD, gene mutations act not only as the core engines driving malignant transformation but also as critical variables determining tumor heterogeneity and clinical outcomes. As the most prominent driver gene in LUAD, the mutation status of EGFR has become the gold standard for defining molecular subtypes and guiding targeted therapies. However, focusing solely on single mutation sites fails to fully elucidate the complex behaviors of tumors regarding proliferation, metabolic reprogramming, and immune evasion. Therefore, leveraging bioinformatics approaches to mine key genes highly correlated with EGFR mutations at the whole‐genome level will not only help decode the systemic evolution of LUAD molecular regulatory networks but also provide precise molecular targets for developing more robust multitarget combination therapies and prognostic evaluation models. Using the Lung Adenocarcinoma Met Organotropism (MSK, Cancer Cell 2023) dataset from the cBioPortal database, we analyzed the EGFR mutation frequency, which showed an overall mutation rate of 35% (Figure 1A). Subsequent survival analyses were performed to compare overall survival (OS) between EGFR‐mutant and wild‐type groups, as well as to compare OS between patients with versus without lymph node metastasis (stratified by EGFR mutation status) and between patients with versus without bone metastasis (similarly stratified). These analyses consistently demonstrated that the EGFR‐mutant group had a significantly better prognosis than the non‐mutant group (Figure 1B–D). We then divided the cohort based on EGFR mutation status and performed a differential analysis (Figure 1E–F). Setting the screening criteria at an absolute Log2 Ratio > 1 and a *p* value < 0.05, we identified a total of 74 genes with differential mutation frequencies, among which 18 genes exhibited significant value in both expression and prognosis in LUAD. The differential expression and prognostic significance of these 18 genes are visualized via heatmaps and forest plots, respectively (Figure 1G–H). Finally, we illustrated the mutation frequencies of these 18 genes between the EGFR‐mutant and non‐mutant groups (Figure 1I).

### 3.2. Molecular Subtyping Based on Key Mutant Genes

Consensus clustering analysis was performed on the TCGA‐LUAD cohort utilizing the expression profiles of the 18 key mutant‐associated genes and employing the non‐negative matrix factorization (NMF) algorithm. The optimal number of clusters for subsequent investigation was determined using the consensus cumulative distribution function (CDF) as the primary quantitative metric, wherein the initial occurrence of a maximal inflection point (peak) on the CDF curves signifies the ideal cluster count. Although the most pronounced decline in the CDF curve was observed at *k* = 5, stratifying the cohort into five subgroups would engender excessive sample fragmentation, thereby compromising the robustness of downstream analyses and rendering this configuration suboptimal. Accordingly, the cohort was stratified into two, three, and four clusters for comparative evaluation (Figure 2A–B). Survival analysis revealed significant differences in overall survival across the two‐, three‐, and four‐cluster classifications. Specifically, in the two‐ and three‐cluster schemes, patients in Cluster 1 exhibited worse survival outcomes, whereas in the four‐cluster scheme, patients in Cluster 3 had the poorest prognosis (Figure 2C–E). Furthermore, we compared the expression profiles of the 18 key mutant genes across the different subgroups. In the two‐cluster stratification, all genes except NKX3‐1 and KRAS exhibited statistically significant differential expression. In contrast, under the three‐cluster classification, many genes failed to show significant expression differences among the subgroups (Figure 2F–G).

Figure 2Clustering analysis of key genes associated with EGFR mutations. (A) Evaluation of clustering stability and performance using multiple metrics. (B) Consensus matrix visualization of the NMF analysis. (C–E) Differences in survival outcomes among different subgroups. (F–G) Differential expression patterns of EGFR mutation‐related genes across the clusters. ns, *p* > 0.05; ∗*p* < 0.05; ∗∗*p* < 0.01; ∗∗∗*p* < 0.001.

**Figure:**
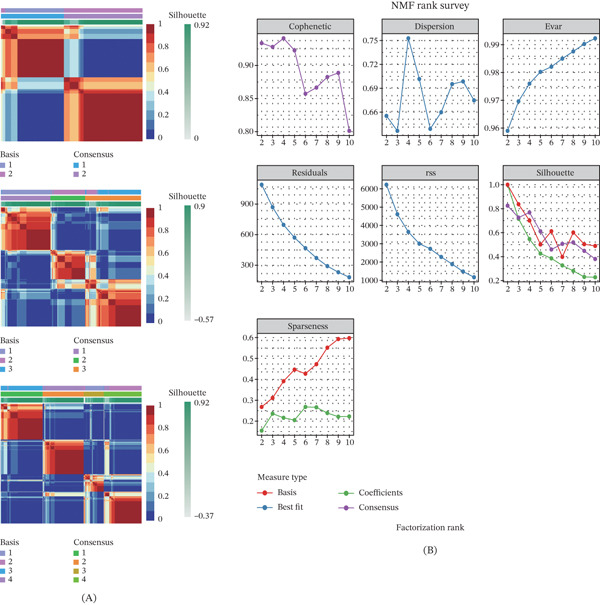
(A)

**Figure:**
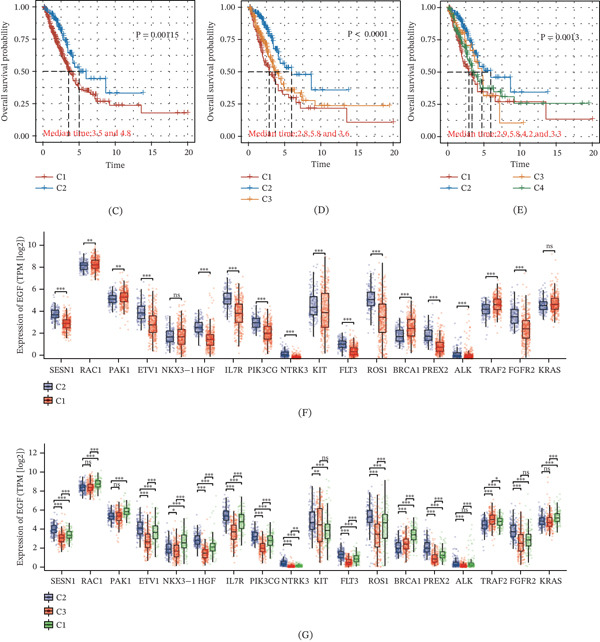
(B)

### 3.3. Correlation Analysis Between EGFR Mutation‐Related Genes and Immunotherapy

Based on the aforementioned findings, we chose to stratify the patients into two clusters for subsequent analyses. To evaluate the association between the identified EGFR mutation‐related genes and immunotherapy outcomes in LUAD patients, we utilized the CIBERSORT algorithm to quantify the infiltration levels of 22 immune cell types across the different cluster subgroups. The results indicated that, compared to Cluster 1, Cluster 2 exhibited significantly elevated infiltration levels of memory B cells, resting memory CD4+ T cells, M2 macrophages, resting myeloid dendritic cells, monocytes, and activated mast cells. Conversely, Cluster 1 showed higher infiltration levels of plasma B cells, CD8+ T cells, activated memory CD4+ T cells, follicular helper T cells, activated NK cells, M0 macrophages, and M1 macrophages than Cluster 2 (Figure 3A–C). Subsequently, we investigated the differential expression of multiple immune checkpoint‐related genes between the subgroups. We found that CD274, CTLA4, HAVCR2, PDCD1, PDCD1LG2, SIGLEC15, ITPRIPL1, and TIGIT were more highly expressed in Cluster 2 (Figure 3D). The Tumor Immune Dysfunction and Exclusion (TIDE) algorithm can predict the clinical response of specific samples or subtypes to immune checkpoint inhibitors. By applying this algorithm, we discovered that patients in Cluster 1 had significantly higher TIDE scores than those in Cluster 2, suggesting that the Cluster 1 group might experience poorer efficacy when receiving immune checkpoint blockade therapy (Figure 3E). To explore the underlying mechanisms driving the immunotherapeutic differences between the clusters, we performed a functional enrichment analysis on each subgroup. The results revealed that the C1 subgroup was predominantly enriched in the cell cycle, DNA replication, glutathione metabolism, biosynthesis of amino acids, and the p53 signaling pathway, whereas the C2 subgroup was mainly enriched in hematopoietic cell lineage, cell adhesion molecules, cytokine–cytokine receptor interaction, intestinal immune network for IgA production, and Th17 cell differentiation (Figure 3F). Finally, we examined the distribution of patients at different clinical stages within the subgroups, demonstrating significant differences between Cluster 1 and Cluster 2 regarding T and N staging (Figure 3G–H).

Figure 3Association of EGFR mutation‐related genes with immune infiltration in LUAD. (A–B) Association between EGFR mutation‐related gene expression and immune infiltration levels analyzed using the CIBERSORT algorithm. (C) Heatmap visualization of immune infiltration profiles between the different subgroups. (D) Differential expression of immune checkpoints between the clusters. (E) Prediction of patient responsiveness to immunotherapy across different clusters using the TIDE algorithm. (F) KEGG analysis of differentially enriched pathways between the clusters. (G–H) Distribution of T stage and N stage populations between the clusters. ns, *p* > 0.05; ∗*p* < 0.05; ∗∗*p* < 0.01; ∗∗∗*p* < 0.001.

**Figure:**
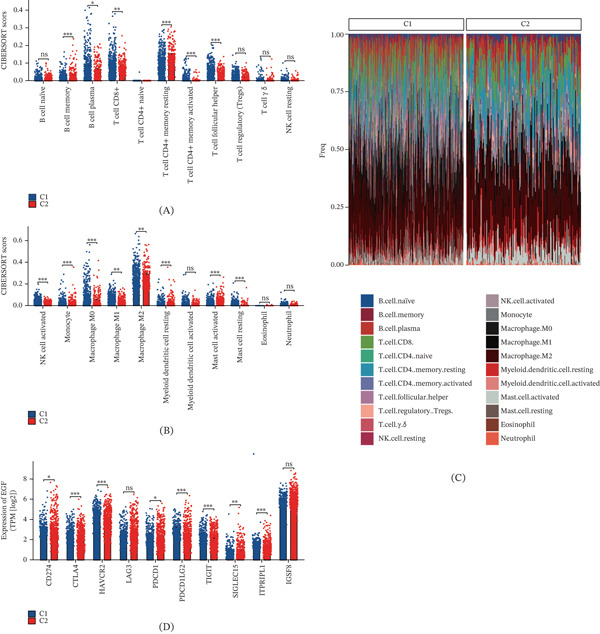
(A)

**Figure:**
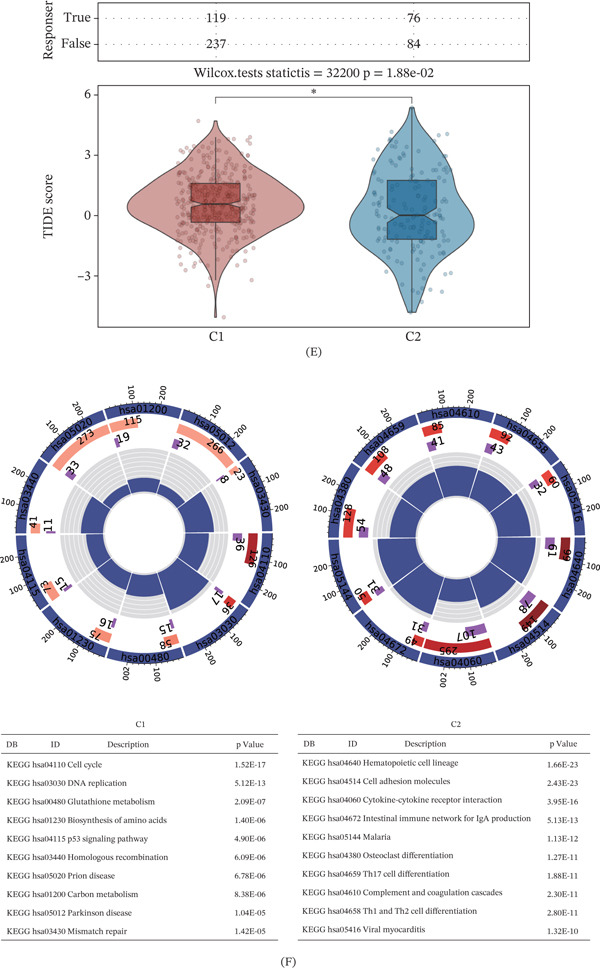
(B)

**Figure:**
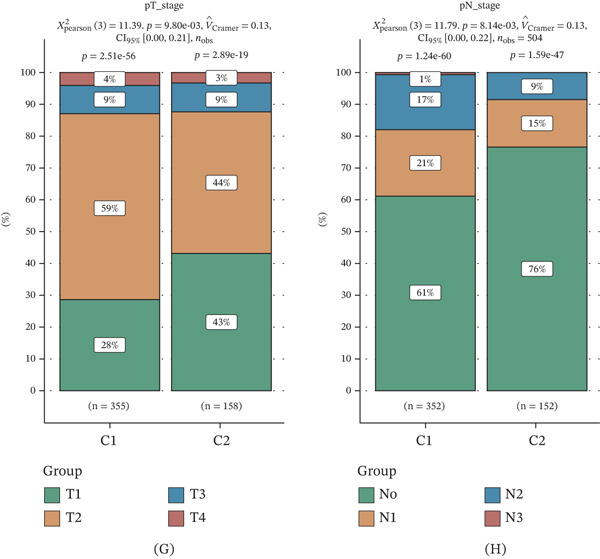
(C)

### 3.4. Correlation Analysis Between EGFR Mutation‐Related Genes and EGFR

**Figure 4:**
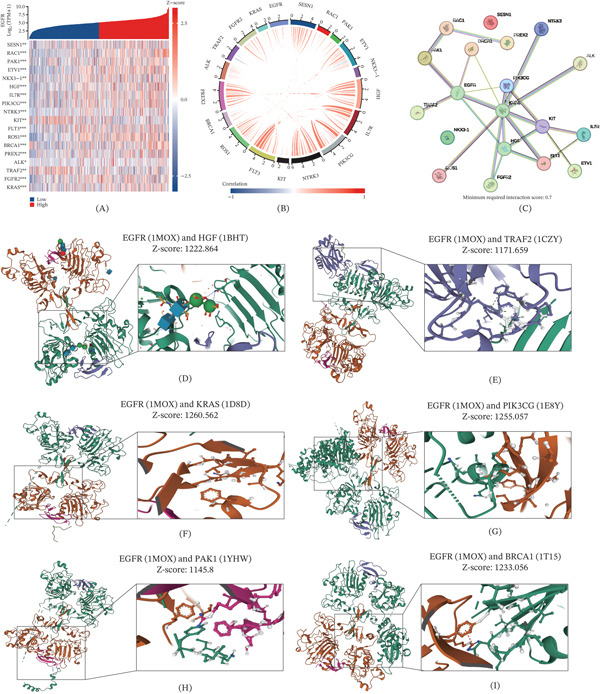
Significant correlations and interactions between EGFR mutation‐related genes and EGFR. (A–B) Correlation analysis between the expression levels of EGFR and the 18 identified genes in the TCGA‐LUAD cohort. (C) STRING database results revealing interactions between six genes and EGFR. (D–I) Molecular docking between EGFR and the six candidate interacting proteins. ns, *p* > 0.05; ∗*p* < 0.05; ∗∗*p* < 0.01; ∗∗∗*p* < 0.001.

To further validate the relationship between the identified EGFR mutation‐related genes and EGFR itself, we analyzed their correlations within the TCGA‐LUAD cohort. The results revealed that EGFR expression was negatively correlated with KIT and TRAF2, whereas it was positively correlated with the remaining 10 genes (Figure 4A–B). Subsequently, we investigated the functional interaction network between these 18 genes and EGFR using the STRING database. The analysis indicated that HGF, TRAF2, KRAS, PIK3CG, PAK1, and BRCA1 possess functional or physical interactions with EGFR (Figure 4C). Moreover, to confirm these interactions from a structural perspective, we performed molecular docking between these six candidate genes and EGFR. The docking results robustly supported the aforementioned findings, demonstrating that each of these six genes exhibits strong binding potential with EGFR (Figure 4D–I).

### 3.5. TRAF2 Is Identified as a Key Prognostic Gene in LUAD

We first utilized the GOsemsim algorithm to prioritize the genes based on functional similarity (Figure 5A). Following this, we employed the XGBoost algorithm to rank the importance of the EGFR‐related genes according to the OS outcomes of the patients (Figure 5B). We also evaluated the prognostic significance of these genes through a multivariate Cox regression analysis, all of which pointed to TRAF2 as a critical prognostic gene (Figure 5C–D). Although TRAF2 was not ranked as the top gene based on the results in Figure 5A, B, both the multivariate Cox regression analysis and the molecular docking results (Figure 4E) revealed that TRAF2 not only maintains a crucial interaction with EGFR but also obtained the highest molecular docking score among the factors significant in the multivariate analysis. Subsequently, we stratified the TCGA‐LUAD cohort based on TRAF2 expression levels and analyzed the distribution of patients across various clinical parameters. The findings indicated that TRAF2 expression is significantly associated with patient age, gender, clinical outcomes, and smoking status (Figure 5E–H). We further explored the relationship between TRAF2 and the LUAD immune microenvironment, uncovering significant correlations between TRAF2 expression and the infiltration levels of 17 different immune cell types (Figure 5I–J). Finally, we analyzed the biological functions of TRAF2 in LUAD, showing that it is heavily implicated in apoptosis, tumor proliferation, DNA repair, and the p53 signaling pathway (Figure 5K).

Figure 5TRAF2 is identified as a key gene associated with EGFR mutations. (A) Gene prioritization based on the GOsemsim algorithm. (B) Identification of key genes related to OS in LUAD using the XGBoost algorithm. (C–D) Multivariate Cox regression analysis of the key genes. (E–H) Correlation analysis between TRAF2 expression and different clinical variables. (I–J) Correlation analysis between TRAF2 expression and immune cell infiltration. (K) Functional analysis of TRAF2 in LUAD. ns, *p* > 0.05; ∗*p* < 0.05; ∗∗*p* < 0.01; ∗∗∗*p* < 0.001.

**Figure:**
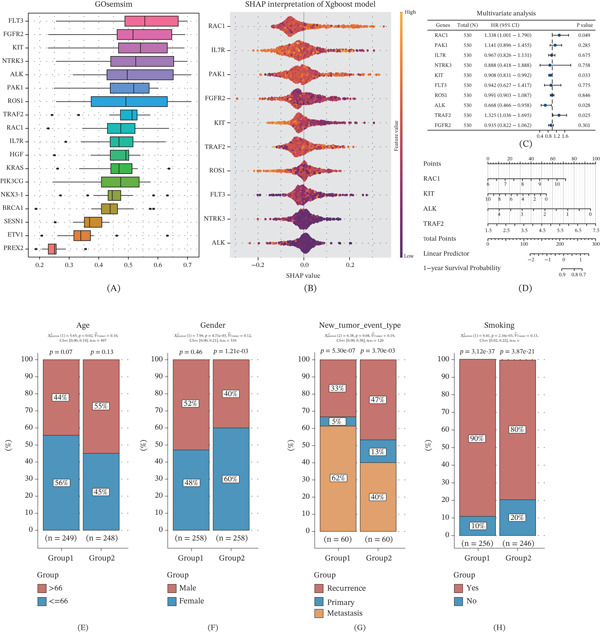
(A)

**Figure:**
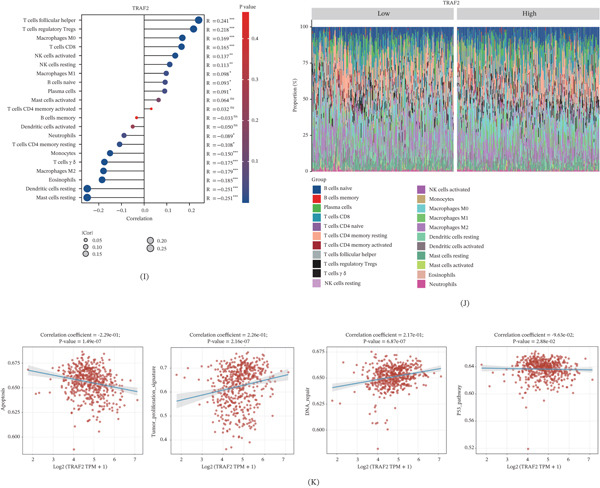
(B)

### 3.6. Expression and Functional Analysis of TRAF2

**Figure 6:**
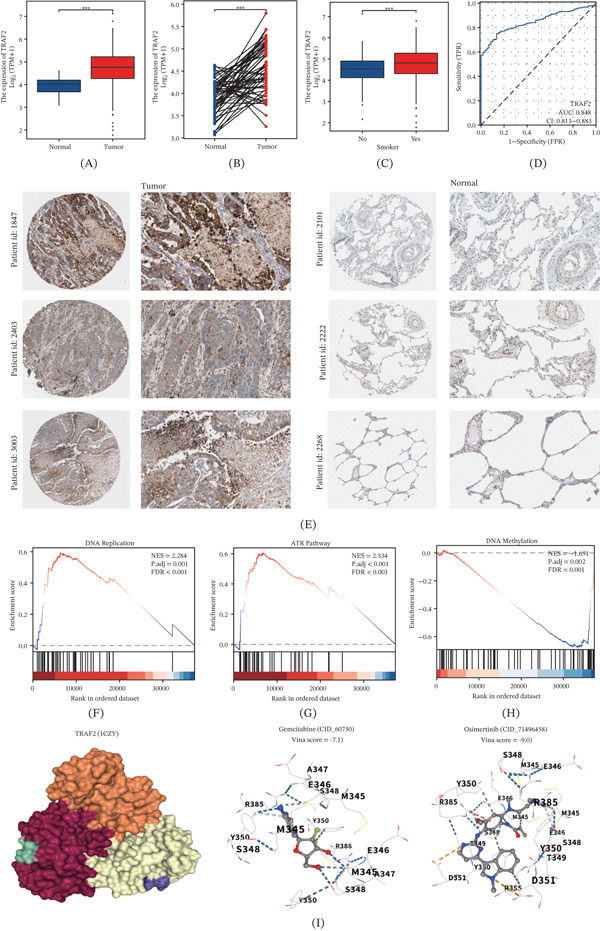
TRAF2 is highly expressed in LUAD. (A–B) Differential expression of TRAF2 between LUAD and normal samples. (C) Differential expression of TRAF2 between smoking and nonsmoking LUAD samples. (D) Diagnostic value of TRAF2 in LUAD. (E) Differential expression of TRAF2 protein between lung cancer and normal tissues. (F–H) Gene set enrichment analysis. (I) Molecular docking of TRAF2 with therapeutic drugs.

By analyzing the expression differences in paired and unpaired samples from the TCGA‐LUAD dataset, we confirmed that TRAF2 is significantly upregulated in LUAD (Figure 6A–B). Furthermore, it was found that the expression level of TRAF2 was significantly higher in LUAD patients with a history of smoking compared to nonsmokers (Figure 6C). We also assessed the diagnostic value of TRAF2 expression in LUAD patients, with ROC curves demonstrating its robust diagnostic potential (Figure 6D). Analysis from The Human Protein Atlas (HPA) database revealed that TRAF2 protein levels were markedly elevated in LUAD tissues relative to normal lung tissues (Figure 6E). Gene set enrichment analysis (GSEA) indicated that TRAF2 is predominantly associated with DNA methylation, the ATR signaling pathway, and DNA replication in lung cancer (Figure 6F–H). Finally, we evaluated the binding affinity of TRAF2 with common therapeutic drugs for LUAD; molecular docking results demonstrated that TRAF2 possesses strong binding affinities for both gemcitabine and osimertinib (Figure 6I).

### 3.7. Analysis of TRAF2 Methylation Levels

**Figure 7:**
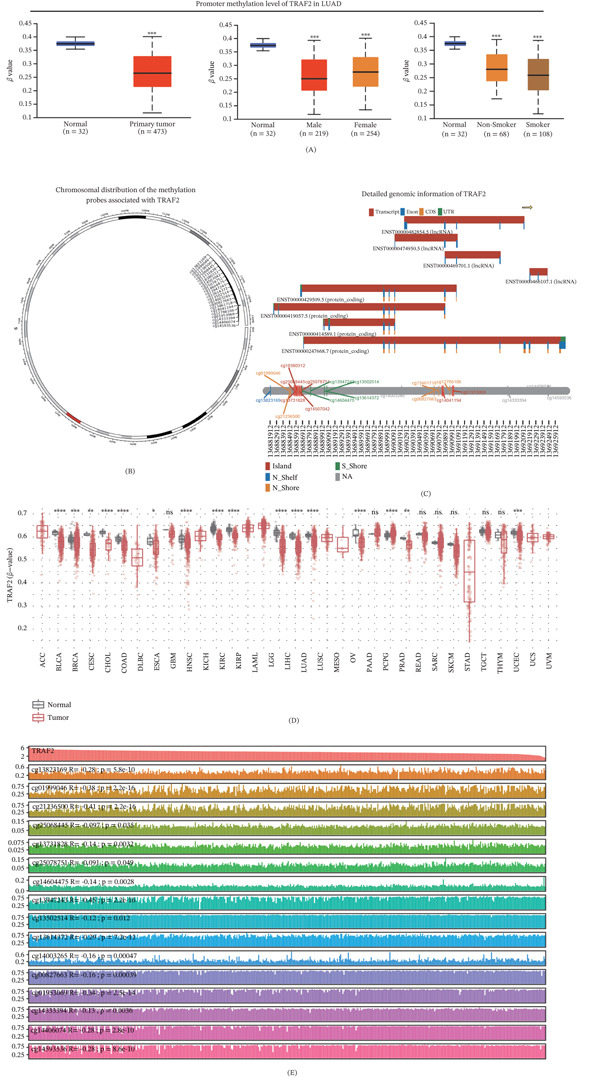
The methylation level of TRAF2 is significantly lower in LUAD than in normal samples. (A) Comparison of TRAF2 DNA methylation levels across different LUAD‐related clinical factors. (B) Chromosomal distribution analysis of TRAF2‐associated methylation probes. (C) Detailed genomic information regarding TRAF2. (D) Analysis of TRAF2 methylation levels across various tumor types. (E) Correlation analysis between TRAF2‐related methylation probes and TRAF2 expression in LUAD. ns, *p* > 0.05; ∗*p* < 0.05; ∗∗*p* < 0.01; ∗∗∗*p* < 0.001.

The functional enrichment analysis of TRAF2 revealed a close association with DNA methylation in LUAD. To deeply understand this relationship, we conducted an in‐depth investigation into the DNA methylation levels of TRAF2 in LUAD. Our research demonstrated that the DNA methylation levels of TRAF2 in LUAD samples were significantly lower than those in normal lung samples. Notably, the methylation levels of TRAF2 also exhibited significant differences between males and females, as well as between smoking and nonsmoking groups (Figure 7A). To further elucidate the role of TRAF2 methylation in LUAD, we queried the SMART database. Initially, we mapped the chromosomal distribution of TRAF2‐related methylation probes in LUAD, followed by an analysis of extensive genomic information associated with TRAF2 (Figure 7B–C). We also presented the variations in TRAF2 methylation levels across different tumor types (Figure 7D). Finally, we explored the correlation between these methylation probes and TRAF2 expression in LUAD, observing that 16 methylation probes were negatively correlated with TRAF2 expression (Figure 7E). In summary, we analyzed the correlations between TRAF2 and various methylation probes in LUAD, proposing that TRAF2 may exert its oncogenic effects in LUAD through these specific methylation sites.

### 3.8. Knockdown of TRAF2 Inhibits LUAD Cell Proliferation and Metastasis

We employed qRT‐PCR technology to validate the knockdown efficiency of different TRAF2 siRNA target sites. The results demonstrated that siTRAF2#1 and siTRAF2#2 exhibited the most significant silencing effects (Figure 8A), and these two targets were therefore selected for subsequent in vitro experiments. In LUAD cell lines A549 and NCI‐H1299, we first analyzed the regulatory role of TRAF2 in the metastatic potential of LUAD cells through wound healing assays. Our findings revealed that the migration ability of LUAD cells was significantly reduced after TRAF2 knockdown (Figure 8B–D). Subsequently, we analyzed the regulatory effect of TRAF2 on the proliferative capacity of LUAD cells through colony formation assays and observed that knockdown of TRAF2 significantly inhibited cell proliferation (Figure 8E–F). Finally, Transwell assay results demonstrated that silencing TRAF2 effectively attenuated the migration and invasion abilities of A549 and NCI‐H1299 cells (Figure 8G–J).

Figure 8Suppressing TRAF2 expression inhibits the growth and metastasis of lung cancer cells. (A) Evaluation of the silencing efficacy of TRAF2 siRNAs via qRT‐PCR. (B–D) Detection of the effect of TRAF2 knockdown on cell migratory ability via wound‐healing assays. (E–F) Assessment of the impact of TRAF2 knockdown on cell proliferation capacity using colony formation assays. (G–J) Determination of the influence of TRAF2 knockdown on cell invasive capacity using Transwell invasion assays.

**Figure:**
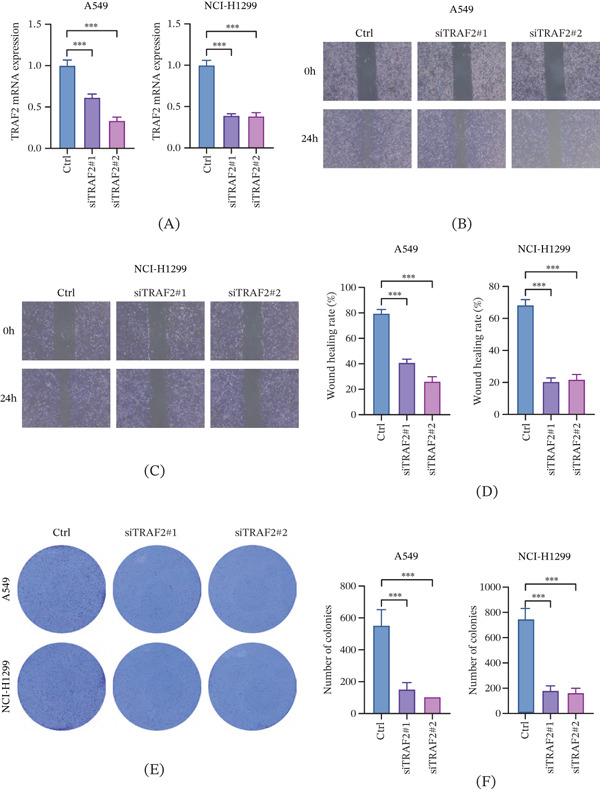
(A)

**Figure:**
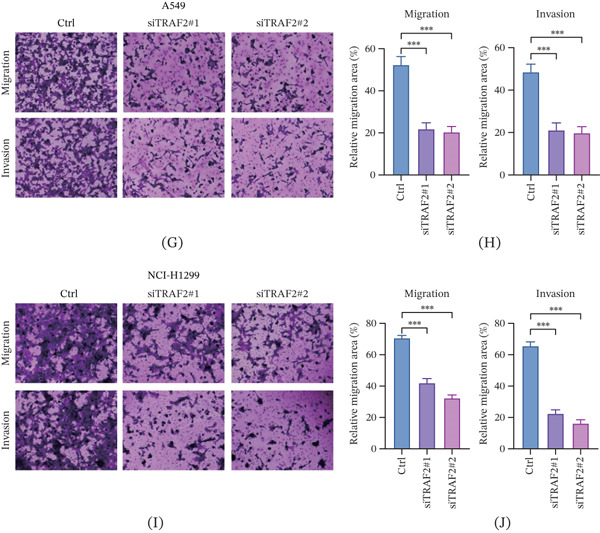
(B)

## 4. Discussion

LUAD, one of the leading causes of cancer‐related mortality worldwide, is characterized by pronounced molecular heterogeneity, which is a key factor contributing to therapeutic resistance and poor prognosis [26]. Although significant progress has been made in targeted therapies against EGFR mutations, the complexity of the TME and the presence of multigene cooperative alterations render single‐mutation detection insufficient for precise prognostic prediction. In this study, bioinformatics approaches were employed to systematically identify 18 core genes highly associated with EGFR mutations and patient prognosis. Based on these genes, novel molecular subtypes were constructed, providing new insights into the systemic evolution of LUAD and advancing strategies for precision clinical treatment.

In the present study, we integrated multi‐omics data from cBioPortal and TCGA‐LUAD to identify 18 EGFR mutation‐associated core genes and established a two‐cluster molecular classification that effectively stratifies LUAD patients by prognosis and immune microenvironment features. Notably, Cluster 2 exhibited higher expression of multiple immune checkpoints (CD274, CTLA4, PDCD1, and TIGIT) and lower TIDE scores, suggesting a potentially better response to immune checkpoint blockade. This finding contrasts with the conventional view that EGFR‐mutant LUAD is uniformly an “immune desert.” Our data indicate that within EGFR‐mutant tumors, there exists considerable immunogenic heterogeneity that can be captured by the 18‐gene signature, which may help guide personalized immunotherapy decisions. Among the 18 core genes identified, TRAF2 was prioritized by multivariate Cox regression, GOsemSim semantic similarity analysis, and the XGBoost algorithm as the most prognostically consequential gene exhibiting direct interaction with EGFR. The inverse correlation observed between EGFR and TRAF2 expression within the TCGA cohort, in conjunction with the robust binding affinity corroborated by molecular docking simulations, intimates the existence of a previously underappreciated reciprocal regulatory axis. Although TRAF2 is canonically recognized as an adaptor molecule within tumor necrosis factor (TNF) receptor signaling cascades, its physical and functional engagement with receptor tyrosine kinases, particularly EGFR, has seldom been documented. The current investigation provides foundational computational and experimental evidence supporting the premise that TRAF2 may function as a downstream mediator or a context‐dependent modulator of EGFR‐directed signaling in LUAD. Functional experiments demonstrated that siRNA‐mediated TRAF2 knockdown significantly suppressed proliferation, migration, and invasion in both A549 and NCI‐H1299 LUAD cell lines. These results are consistent with a growing body of evidence implicating TRAF2 in tumor progression across multiple cancer types. In lung cancer, recent reports indicate that TRAF2 can be activated by KEAP1 mutations to enhance NF‐*κ*B–dependent anti‐apoptotic signaling and that MYSM1 stabilizes TRAF2 to sustain noncanonical NF‐*κ*B activation [32, 33]. Our GSEA further revealed that TRAF2 expression is tightly associated with DNA methylation, ATR signaling, and DNA replication pathways, suggesting that TRAF2 may contribute to LUAD aggressiveness by promoting genomic instability and replicative stress tolerance.

DNA methylation analysis revealed that TRAF2 is significantly hypomethylated in LUAD tissues compared with normal lung samples, and 16 methylation probes showed a negative correlation with TRAF2 expression. This epigenetic upregulation mechanism is reminiscent of findings in gastric cancer, where TRAF2 hypomethylation serves as an independent poor prognostic indicator [34]. Importantly, we observed differential TRAF2 methylation between sexes and smoking statuses, hinting that environmental and host factors may modulate TRAF2 expression through epigenetic reprogramming. These observations position TRAF2 as a potential bridge between external exposures and the intrinsic EGFR‐driven oncogenic network in LUAD. The molecular docking results further indicated that TRAF2 possesses strong binding affinities for gemcitabine and osimertinib, two drugs commonly used in LUAD treatment. Although the functional significance of these interactions requires experimental validation, they raise the intriguing possibility that TRAF2 could directly influence drug efficacy or serve as a pharmacodynamic biomarker.

Several limitations should be considered. First, the functional validation was limited to two LUAD cell lines (A549 and NCI‐H1299). We acknowledge that A549 cells harbor a KRAS mutation and NCI‐H1299 cells lack EGFR protein expression; thus, while these lines allowed us to demonstrate a general protumorigenic role of TRAF2, the specificity of TRAF2 function within the EGFR‐mutant axis remains indirect. Therefore, the results need to be extended to additional models, including canonical EGFR‐mutant cell lines (e.g., PC9 or HCC827), patient‐derived organoids, and in vivo xenograft studies, to specifically assess the impact of TRAF2 modulation on EGFR‐TKI sensitivity and EGFR‐driven phenotypes. Second, the exact molecular mechanism by which TRAF2 interacts with EGFR—whether through direct ubiquitination, scaffolding, or transcriptional regulation—remains to be elucidated. Third, the 18‐gene signature and the two‐cluster classification were derived from public databases and require prospective validation in independent clinical cohorts with standardized immunotherapy protocols. Despite these limitations, our study provides several novel insights. We demonstrate that TRAF2 is not only a prognostic marker but also a functional driver of LUAD progression, and we uncover its epigenetic upregulation via hypomethylation. The 18‐gene molecular classification offers a practical tool to dissect the immunogenic heterogeneity within EGFR‐mutant LUAD, potentially guiding combination strategies that pair EGFR‐TKIs with immune checkpoint inhibitors. Future work should focus on developing selective TRAF2 inhibitors and evaluating their therapeutic window in LUAD mouse models, as well as exploring whether TRAF2 expression levels can predict resistance to osimertinib or chemotherapy.

## 5. Conclusion

In summary, by integrating bioinformatics, molecular docking, and in vitro experiments, we have identified TRAF2 as a key EGFR‐associated prognostic gene in LUAD that promotes tumor proliferation, migration, and invasion. The established 18‐gene molecular classification effectively distinguishes patient subgroups with distinct immune profiles and immunotherapy response potentials. These findings expand the current understanding of the EGFR‐centric regulatory network in LUAD and open new avenues for epigenetic and targeted combination therapies.

## Author Contributions

Bo Gao: original draft, visualization, validation, methodology. Zihan Dong: investigation, formal analysis, conceptualization. Taotao Yan: validation, methodology. Zhiwang Liu: visualization, validation. Jianle Chen: writing – review and editing, supervision, resources, and funding acquisition. Bo Gao and Zihan Dong contributed equally to this work.

## Funding

This research was financially supported by two projects: the Nantong Natural Science Foundation (Grant No. JC2024071) and the Beijing Kangmeng Charity Foundation (Grant No. HXKT20241056).

## Disclosure

All authors have read and approved the final manuscript.

## Ethics Statement

The authors have nothing to report.

## Conflicts of Interest

The authors declare no conflicts of interest.

## Data Availability

The data that support the findings of this study are available from the corresponding author upon reasonable request.
